# How different augmented reality visualizations for drilling affect trajectory deviation, visual attention, and user experience

**DOI:** 10.1007/s11548-022-02819-5

**Published:** 2023-02-18

**Authors:** Julian Wolf, Dietmar Luchmann, Quentin Lohmeyer, Mazda Farshad, Philipp Fürnstahl, Mirko Meboldt

**Affiliations:** 1 PDZ, Department of Mechanical and Process Engineering, ETH Zurich, Zurich, Switzerland; 2 ROCS, Balgrist University Hospital, University of Zurich, Zurich, Switzerland; 3 Department of Orthopaedics, Balgrist University Hospital, Zurich, Switzerland

**Keywords:** Mixed reality, Surgical navigation, Eye tracking, Usability, Cognitive load

## Abstract

**Purpose:**

Previous work has demonstrated the high accuracy of augmented reality (AR) head-mounted displays for pedicle screw placement in spinal fusion surgery. An important question that remains unanswered is how pedicle screw trajectories should be visualized in AR to best assist the surgeon.

**Methodology:**

We compared five AR visualizations displaying the drill trajectory via Microsoft HoloLens 2 with different configurations of abstraction level (abstract or anatomical), position (overlay or small offset), and dimensionality (2D or 3D) against standard navigation on an external screen. We tested these visualizations in a study with 4 expert surgeons and 10 novices (residents in orthopedic surgery) on lumbar spine models covered by Plasticine. We assessed trajectory deviations ($^\circ$) from the preoperative plan, dwell times (%) on areas of interest, and the user experience.

**Results:**

Two AR visualizations resulted in significantly lower trajectory deviations (mixed-effects ANOVA, *p*<0.0001 and *p*<0.05) compared to standard navigation, whereas no significant differences were found between participant groups. The best ratings for ease of use and cognitive load were obtained with an abstract visualization displayed peripherally around the entry point and with a 3D anatomical visualization displayed with some offset. For visualizations displayed with some offset, participants spent on average only 20% of their time examining the entry point area.

**Conclusion:**

Our results show that real-time feedback provided by navigation can level task performance between experts and novices, and that the design of a visualization has a significant impact on task performance, visual attention, and user experience. Both abstract and anatomical visualizations can be suitable for navigation when not directly occluding the execution area. Our results shed light on how AR visualizations guide visual attention and the benefits of anchoring information in the peripheral field around the entry point.

## Introduction

Spinal fusion surgery is indicated by severe spine disorders and has seen an increase in performed surgeries of up to 200% in the last 30 years [14]. Due to the proximity of vital structures, strong intraoperative bleeding and variability in morphology between patients [2], spinal fusion surgery is a very demanding procedure, resulting in enormous health care costs [10].

Free-hand surgery aided by 2D fluoroscopy is currently the most used approach in spinal fusion surgery, acting as the standard that more recent navigation systems are often compared with [11]. Optical navigation systems (fluoroscopy, CT-guided navigation) were shown to be advantageous in accuracy and radiation exposure compared to free-hand execution or in combination with conventional visualizations [11, 16]. As Härtl et al. have shown in 2013, only a minor part of surgeons utilize these new navigation technologies on a regular basis [5]. Factors stated are prolonged operating room (OR) times, a lack in ease of use and integration into the surgical workflow, and the high cost [5, 13]. Augmented reality (AR) head-mounted displays (HMDs) promise to offer a range of benefits compared to conventional navigation systems [9, 17], such as increased anatomical understanding, execution speed and ease of use, and have seen growing interest over the last years [3]. By superimposing images into the field of view, the operator does not lose sight of the patient by gazing off at monitors [7].

While previous work on AR navigation has demonstrated the high accuracy of AR HMDs for pedicle screw placement, both in simulated [6, 12] and real interventions [4, 6], an important question that remains unanswered is how pedicle screw trajectories should be visualized in AR to best assist the surgeon. Outside of AR, Brendle et al. [1] compared a hand-held navigation device with an integrated circular display showing different abstract visualizations for pedicle screw trajectories against conventional navigation displayed on an external screen. They found a significant reduction (*p*<0.05, Kruskal–Wallis test) in cognitive load and a significantly better usability when operating with the hand-held device. Using an AR HMD instead of a hand-held device, we are not limited to displaying information on the screen space of a display. Instead, we can anchor our AR interfaces in 3D space and, for example, display information in the peripheral area around the tool entry point. Moreover, augmented reality offers a variety of possibilities to display information, ranging from overlay to displaying information next to the patient, from 2D to 3D, and from abstract to anatomical representations. These configurations are expected to not only affect the surgical outcome, but also the user experience and the visual behavior, and are, thus, expected to greatly impact user acceptance. In this paper, we compare five different augmented reality visualizations for pre-drilling pedicle screw trajectories with variations in abstraction level (abstract or anatomical), dimensionality (2D or 3D), and position (overlay or small offset) against conventional navigation on an external screen. We test these visualizations on a (L1-L5) lumbar spine model setup with 4 expert surgeons and 10 novices (residents in orthopedic surgery). We measure and evaluate the trajectory deviation between planned and realized trajectory ($^\circ$) as a metric for task performance, the dwell time (%) on areas of interest (AOIs) as a metric of visual attention, and the user experience with emphasis on ease of use and cognitive load. While our study is concerned with pedicle screw placement, we expect our findings to generalize to other AR-guided orthopedic interventions that are performed on partly occluded anatomy and that require a highly accurate execution.

Contrary to the outcome of free-hand execution, which has been shown to be significantly affected by the surgeons’ experience (*p*<0.01) [8, 15], we expect AR navigation to level the task performance between expert and novice groups. We further expect abstract visualizations to result in the lowest trajectory deviations and best user experience due to the simplified information representation.

## Related work

Liebmann et al. [9] developed an AR navigation for pedicle screw placement with Microsoft HoloLens. Their AR interface superimposed the planned trajectory and the tool trajectory. The ends of both trajectories were connected by a line and the numeric trajectory deviation was displayed on top of the tool marker. We use the same combination of planned trajectory, tool trajectory and numeric trajectory deviation display for all our anatomical visualizations.

In a study similar to ours, Brendle et al. [1] compared a hand-held navigation device for pedicle screw placement with conventional navigation displayed on an external screen. Their hand-held device comprised of a drill sleeve with build-in circular display that showed the trajectory deviation in two different abstract visualizations. The first visualization, the ‘circle display’, shows the tool trajectory as a point moving continuously across the underlying background. The target trajectory is achieved by moving the point into the center of the display. The ‘grid display’ divides the circular interface into 12 pie sections and four concentric circles of different radii. According to the relative orientation of the tool toward the planned trajectory, the respective grid field is highlighted in red, with the center field representing the target orientation. As part of our study, we also investigate two abstract visualizations, one with a continuous angle display like the ‘circular display’, and one using discrete ring segments like the ‘grid display’.

**Fig. 1:**
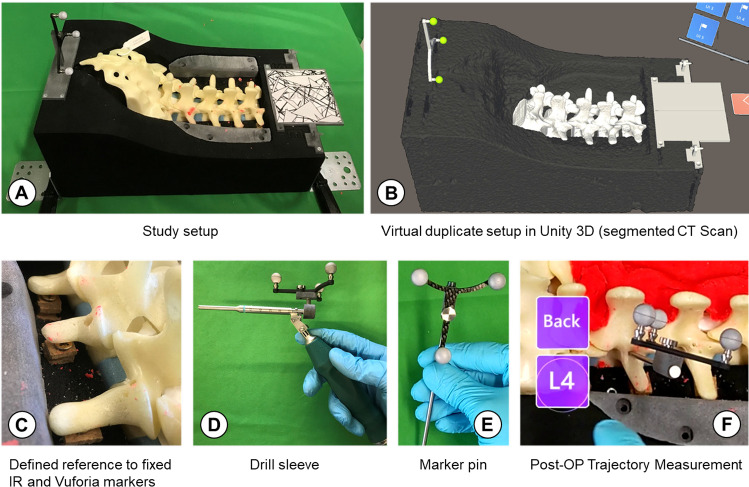
Physical setup (**A**) with L1-L5 lumbar spine model in spine bed and virtual duplicate setup (**B**). The physical setup uses a fixed IR marker as a reference for tool tracking and a Vuforia image marker for initial registration with Microsoft HoloLens 2. The spine bed was reinforced with wooden pads (**C**) to fully constrain relative movement between spine and spine bed. The setup uses a drill sleeve for navigation (**D**) and a marker pin (**E**) for post-OP trajectory measurement (**F**). The latter is done by partly removing the Plasticine, inserting the marker pin into the drilled pedicle channel, and saving its 3D pose by pressing a button in a graphical user interface

## Materials and methods

### Apparatus

**Fig. 2:**
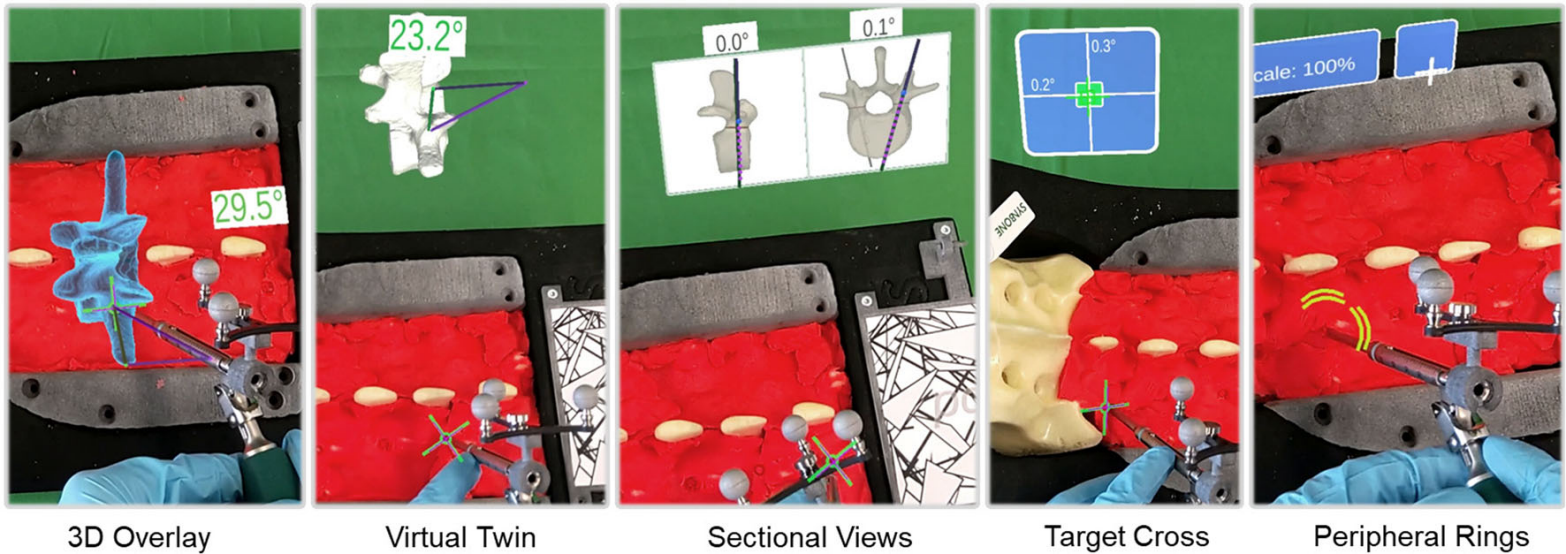
All five AR visualizations shown from the participants’ point of view. The spine is covered by red Plasticine

The AR navigation app was implemented for Microsoft HoloLens 2 using Unity 3D (2019.4.14f1) and the Mixed Reality Toolkit (MRTK 2.4.0). The standard navigation app was implemented as a desktop app using the same Unity 3D backend. The simulator setup (cf. Fig. 1) was based on a spine bed for a L1-L5 lumbar spine model (Synbone AG, Zizers, Switzerland) and had both a Vuforia image marker attached for initial registration with HoloLens 2 and a fixed infrared (IR) marker as a reference for tool tracking. The spine was covered with red Plasticine (best visible in Fig. 2) to increase difficulty and thus the need for navigation. The tracking camera (Atracsys LLC, Puidoux, Switzerland; not visible in Fig. 1) was connected via cable to a desktop computer, running a server application that streamed incoming data points (i.e., transformation matrices of detected IR markers) to the client application (either to a Unity desktop app or to HoloLens 2). Three different tools were tracked by the external camera: a drill sleeve for navigation, a marker metal pin that can be inserted into the drilled pedicle channels to perform a post-op trajectory measurement, and a pointer marker for landmark registration of the spine model.

### Outcome parameters

We measured the absolute trajectory deviation (TD) between planned ($\vec {A}$) and executed trajectory ($\vec {B}$) and preoperative plan in radial degrees (cf. Eq. (1)), dwell times on areas of interest in percent (cf. Sect. 3.6), and the user experience with emphasis on visualization ranking, ease of use, and cognitive load. Trajectory deviation measurements were conducted by inserting the marker metal pin into the drilled pedicle channels (cf. Fig. 1). This study was only concerned with trajectory alignment and did not investigate the accuracy of entry point placement.

1 $$\begin{aligned} TD = \cos ^{-1}{\left( \frac{\vec {A} \cdot \vec {B}}{\Vert \vec {A}\Vert \Vert \vec {B}\Vert }\right) } \end{aligned}$$

### Measurement system

#### Tracking system

An Atracsys fusionTrack250 (Atracsys LLC, Puidoux, Switzerland) with an accuracy of 0.09 mm (RMS) within 1.4 m distance was used. To determine the trajectory error between drill sleeve and marker pin, we fixated the drill sleeve in a vice, drilled ideal trajectories at low drilling speed, and subsequently measured the trajectory with the marker pin. We found a trajectory error between drill sleeve trajectory and marker pin trajectory of up to $\pm 0.17$ $^\circ$.

#### Eye tracking

HoloLens 2 reports the wearer’s eye-gaze with an angular accuracy of 1.5$^\circ$ around the actual target and a recording rate of 30 fps.

**Table 1:** Configurations of abstraction level (abstract or anatomical), position (overlay or small offset), and dimensionality (2D or 3D) used to derive the AR visualizations and the standard navigation

Visualization concept	Abstraction level	Position	Dimensionality	Device
‘Standard Navigation’	Anatomical	Large offset	2D	External screen
‘3D Overlay’	Anatomical	Overlay	3D	HoloLens 2
‘Virtual Twin’	Anatomical	Small offset	3D	HoloLens 2
‘Sectional Views’	Anatomical	Small offset	2D	HoloLens 2
‘Target Cross’	Abstract	Small offset	2D	HoloLens 2
‘Peripheral Rings’	Abstract	Overlay	2D	HoloLens 2

#### Questionnaire

The questionnaire consisted of one question on ease of use (cf. Q1) and a NASA-TLX questionnaire for cognitive load (Q2-7) with point ratings between 1 (very difficult/low) and 10 (very easy/high) that was filled out for each visualization. At the end of the experiment, participants were asked to rate the AR visualizations according to their preferences (cf. Q8), with the most preferred visualization scoring 5 points and the least preferred scoring 1 point.

Q 1: How easy was it to drill the intended trajectory with the visualization?
Q 2-7: NASA-TLX questionnaire for cognitive load.
Q 8: Please rank the AR visualizations according to your preference from best to worst.

### Visualizations

From the eight possible configurations of abstraction level, position, and dimensionality, five were considered most useful and implemented (cf. Table 1). Intuitively, we excluded a 2D anatomical overlay as we did not see benefits compared to a 3D overlay. We further excluded 3D abstract visualizations as trajectory deviations along sagittal and transverse planes can be well displayed using two dimensions.

Figure 2 shows the implemented AR visualizations. The planned entry point position is highlighted by a green cross, while the current position of the drill sleeve tip, referred to as tool tip, is displayed as a purple cross. Participants can first position the tool tip on the entry point and then use the respective AR visualization to align the tool trajectory. All (AR and non-AR) visualizations are explained in the following.

#### Standard navigation

**Fig. 3:**
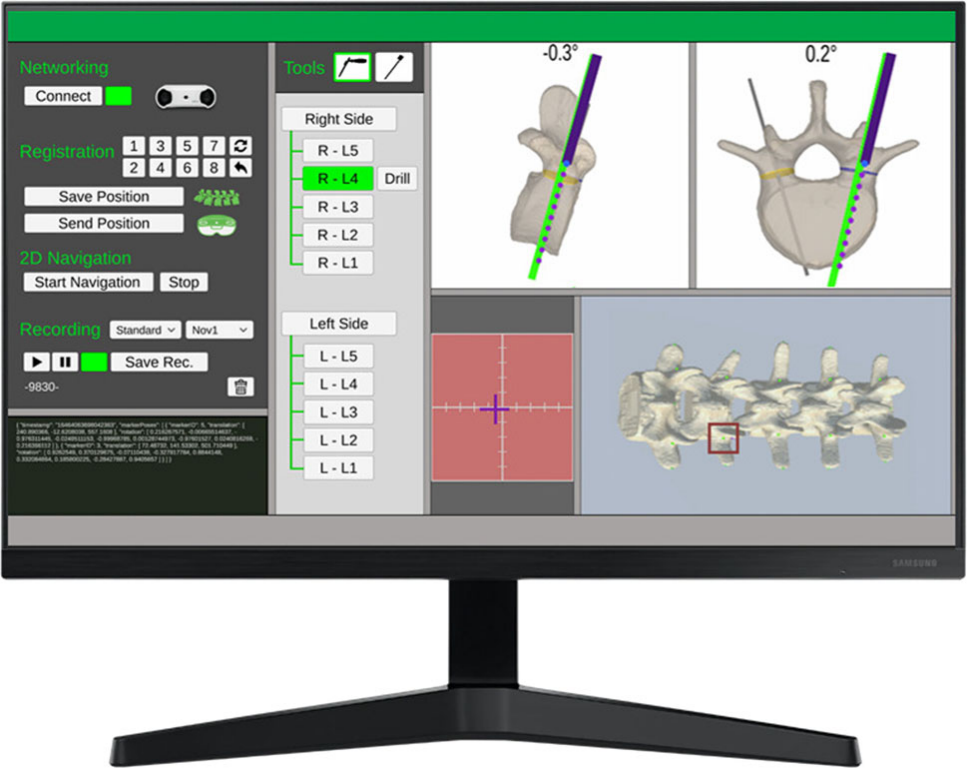
Standard navigation with user interface to connect to the external tracking camera, start recordings and select a vertebrae (left), a top view on the segmented spine model (bottom right), a magnifying view for fine-positioning of the tool tip on the entry point (red area in the middle), and two ‘sectional views’ in sagittal and transverse direction (top right)

This non-AR navigation (cf. Fig. 3) displays sagittal and transverse slices of a segmented CT-model with planned (green) and current tool trajectories (purple) on an external screen and is used as the gold standard.

#### 3D overlay

This AR interface superimposes the whole virtual vertebrae on top of the real one including planned and current trajectories and the absolute numeric deviation.

#### Virtual twin

This AR interface displays the same information as the 3D overlay next to the spine to not occlude the entry point and the tool tip. As a consequence, the surgeon is not disturbed while operating but can countercheck the execution against the virtual model.

#### Sectional views

This AR interface aims to provide a more familiar navigational display that shows the same two sectional views used in the standard navigation, but closer to the entry point.

#### Target cross

This AR interface aims at displaying directional trajectory deviations in an simpler way than the two sectional views by visualizing the trajectory as a red target cross moving continuously on a blue plane. The horizontal axis represents the deviations along the sagittal plane and the vertical axis represents the deviations along the transverse plane. The center area of the blue plane and the red target cross turn green while navigating within 2$^\circ$ deviation.

#### Peripheral rings

This AR interface aims at displaying the same abstract information as the target cross in the peripheral field of the entry point, thus allowing the participant to focus on the entry point without occluding it. Each ring segment represents an angular deviation of 1.41$^\circ$ in the respective direction. If no ring segments are visible, the trajectory lies within the limits of < 2$^\circ$ deviation.

### Study design

The study was divided into an initial testing phase to familiarize participants with the visualizations and a main study. Within the main study, participants pre-drilled 35 pedicle screw trajectories (equals 3.5 L1-L5 lumbar spine models), of which 10 trajectories were drilled using standard navigation and 5 with each of the five AR visualizations. Participants were instructed to drill pedicle screw trajectories with less than 2$^\circ$ deviation from the preoperative plan to create a challenging and immersive task. We derived a study protocol with configurations of visualization types and operation side so that all visualizations were performed an equal number of times on the right and left side by each group. Participants were randomly assigned to these configurations.

#### Participants

We recruited 14 participants, including four expert surgeons (aged 35–42 years) and ten residents in orthopedic surgery (aged 25–36 years).

#### Experimental procedure

**Fig. 4:**
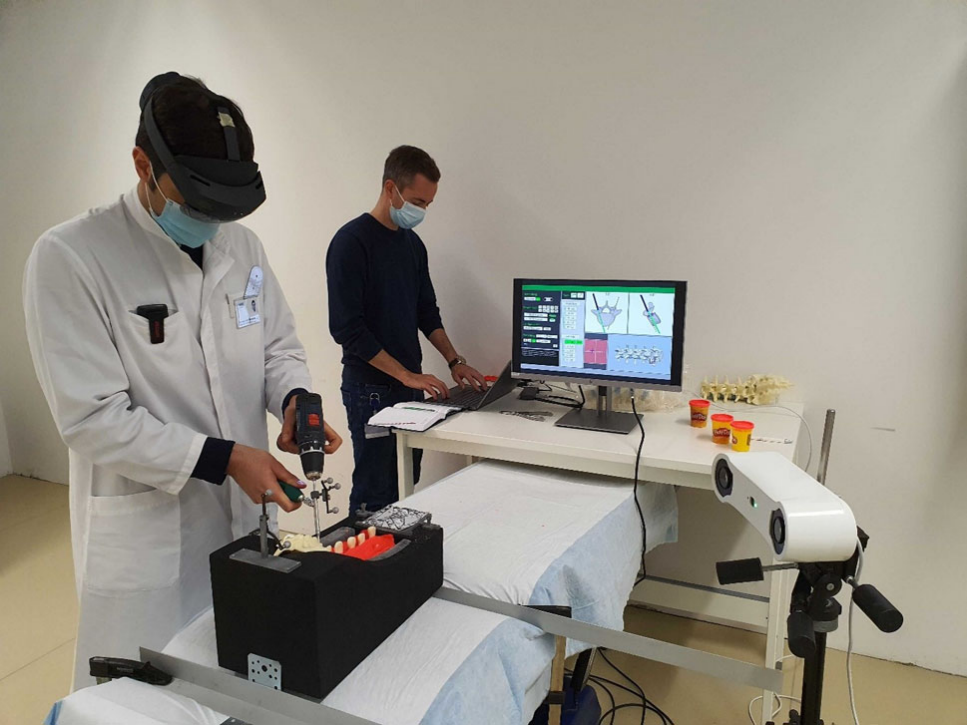
Experimental setup showing the participant navigating with HoloLens 2, the spine bed, tracking camera and external screen

Figure 4 shows the experimental setup used during the main study. Prior to starting with the experiment, participants received a reintroduction to HoloLens 2 and were guided through the calibration procedure of the eye tracking system, which is an automated routine provided by HoloLens 2. Participants then calibrated HoloLens 2 to the experimental setup by confirming the position of the Vuforia image marker. After pre-drilling screw trajectories with each visualization, participants filled out a user experience questionnaire for the respective visualization. We used this time window to perform the post-op trajectory deviation measurement for each pedicle screw channel. Depending on the operating side specified in the study protocol, we then turned the setup by 180$^\circ$ and/or replaced the spine model, and performed a landmark registration to register the spine position to the spine bed. After the completion of the experiment, participants ranked the visualizations according to their preferences and could describe their impressions during an interview.

### Eye tracking analysis

For quantification of visual attention, we divided the stimuli into five Areas of Interest (AOIs). These AOIs comprise of the ‘entry point’ area, all visualizations displayed with offset to the entry point, and the ‘background’, which represents the remaining space. No separate AOIs were defined for the 3D overlay and the peripheral rings as these visualizations are displayed directly on top of the entry point. The time spent examining each AOI was summed up and divided by the total time spent on the task, resulting in the AOI dwell time (%).

**Fig. 5:**
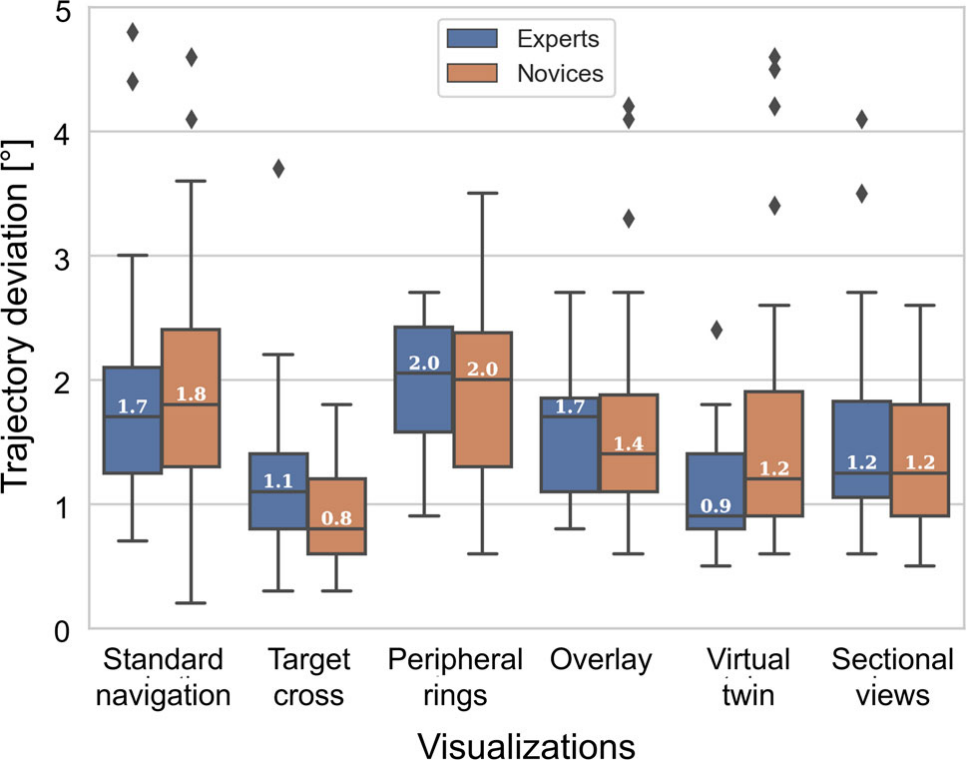
Trajectory deviation measurements over all visualizations for expert (blue, *N* $=$ 4) and novice (brown, *N* $=$ 10) groups

**Table 2:** Relative dwell times (means and stds) on AOI ‘entry point’ and the respective visualization for all time steps with trajectory deviation below 3$^\circ$ both for expert (*N*=4) and novice (*N*=10) groups

Visualization	AOI ‘Entry point’		AOI ‘Visualization’	
	Exp	Nov	Exp	Nov
Target cross	19.5% ± 14.4%	19.2% ± 15.0%	78.3% ± 15.3%	74.9% ± 18.5%
Virtual twin	22.0% ± 16.6%	18.3% ± 14.3%	75.6% ± 17.3%	77.2% ± 16.6%
Sectional views	16.2% ± 10.7%	19.5% ± 16.1%	75.8% ± 15.9%	77.5% ± 17.7%

### Statistical analysis

All statistical tests were conducted using the R environment. We modeled trajectory deviations in a mixed-effects ANOVA that included visualization, skill level, and operator side as fixed effects, and an operator random effect. Each pedicle screw trajectory was considered as one measurement. We tested the residuals of the model for normal distribution and homoscedasticity using the DHARMa package, which did not show any violations and thus confirmed normal distribution. A post hoc analysis was performed with Benjamini–Hochberg adjusted pairwise t tests. Preliminary analysis of questionnaire responses showed them to be undistributed (Shapiro–Wilk Test, *p*<0.05). Questionnaire responses were then analyzed with a Friedman test and a post hoc analysis with pairwise comparisons using the Wilcoxon signed rank test. Participant groups were pooled for statistical analysis of questionnaire responses to account for the small sample sizes.

## Results

### Trajectory deviation

From the 490 pedicle screw trajectories drilled, 10 measurements (2%) were excluded due to problems in data recording with HoloLens 2 and 20 measurements (4%) were excluded as the marker pin could not be fully inserted into the opened pedicle channel. Figure 5 shows the post-op trajectory measurement results. 73% of all measurements lied within the target deviation of 2$^\circ$ and 93% lied within 3$^\circ$ trajectory deviation. The mixed-effects ANOVA with Benjamini–Hochberg adjusted pairwise contrasts showed significant differences when comparing ‘target cross’ with ‘standard navigation’ and ‘peripheral rings’ (both *p*<0.0001), ‘overlay’ (*p*<0.001), ‘virtual twin’ and ‘sectional views’ (both *p*<0.05). We also found significant differences when comparing ‘virtual twin’ against ‘standard navigation’ and ‘peripheral rings’ (both *p*<0.05). We did not observe a significant effect of skill level or operating side on the trajectory deviation (*p*>0.05).

### Visual attention

Table 2 shows the relative dwell times on the AOI ‘entry point’ and all AR visualizations displayed with small offset while trajectory deviations were below 3$^\circ$. Participants spent approximately 20% of their time examining the entry point area and the rest of their time on the respective visualization. Up to 10% of visual attention was usually registered on the AOI ‘background’.

### User experience

Table 3 shows the summary statistics of the questionnaire results. While experts’ preferences of visualizations varied greatly, resulting in overall similar point ratings, novices’ ratings were more determined. The Friedman test using joined participant groups showed significant differences between visualizations for ease of use and cognitive load (*p*<0.01). Post hoc analysis using pairwise Wilcoxon rank tests without p value adjustment method showed significant differences in *ease of use* when comparing ‘peripheral rings’ against ‘standard navigation’, ‘sectional views’, and ‘overlay’ (all *p*<0.01), and smaller differences when comparing ‘target cross’ and ‘virtual twin’ against ‘overlay’ (*p*<0.05). For *cognitive load*, we found statistical differences when comparing ‘peripheral rings’ and ‘virtual twin’ against ‘overlay’ (both *p*<0.01) and against ‘standard navigation’ (both *p*<0.05). No significant differences were found when using the Benjamini–Hochberg procedure for p value adjustment.

**Table 3:**
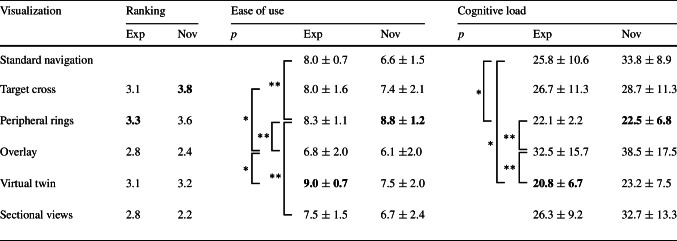
Questionnaire summary results for experts (*N*=4) and novices (*N*=10): visualization point ranking [5 = favorite visualization, 1 = least liked] averaged over participant groups, means, stds, and *p* values for ease of use ([0,10] higher is better) and cognitive load (NASA-TLX, [0, 100] lower is better)

The maximum values of each column are printed in bold

Significance levels: $p\ge 0.05$-NS, *p<0.05, **p<0.01

## Discussion

The goal of this study was to better understand the effects of different AR visualizations for pedicle screw pre-drilling on task performance, visual attention and user experience both for expert surgeons and novice operators. Despite the fact that participants were not trained on the visualizations before the experiment and had previously only explored them, the majority of pedicle screw trajectories was successfully placed within the specified 2$^\circ$ trajectory deviation. The high similarity of outcomes for expert and novice groups indicates that real-time feedback on task execution helps to level task performance.

Although we expected that abstract visualizations would receive the overall best ratings in terms of ease of use and cognitive load, only the peripheral rings were rated significantly better than standard navigation, whereas no such differences were found for the target cross. Participants appreciated that using the peripheral rings they could stay focused on the area of execution. As expected for abstract visualizations, the target cross achieved the best performance in terms of trajectory deviation. An unexpected outcome was the significant difference in trajectory deviation between the target cross and the peripheral rings. Although both of these abstract visualizations show the same target angle of 2$^\circ$, the target cross displays the angle continuously, whereas for the peripheral rings there is a blind spot within 2$^\circ$ range in which the participants do not receive feedback. We believe that by increasing the resolution of the peripheral rings to represent smaller angle increments (e.g., 0.5$^\circ$ increments instead of 1.41$^\circ$), similarly low trajectory deviations are possible.

For anatomical visualizations, we expected the two sectional views to result in the lowest trajectory deviations as deviations can be optimized separately for both directions. Interestingly, participants executed the task more accurately using the virtual twin while both ease of use and cognitive load were much better rated. It seems that multiple anatomical views also increase the difficulty to incorporate this information during task execution.

The anatomical overlay was perceived as overall most distracting and resulted in higher trajectory deviations and worse user experience than the virtual twin. Participants found it irritating that they could not see the tool tip and entry point very well. In contrast, participants found the virtual twin particularly helpful in gaining an understanding of the anatomy and to locate the entry point. Participants further stated that it felt very intuitive that whenever the tool was in contact with the bone, they would also see this contact visually on the virtual twin.

In summary, our results support previous studies that have shown AR navigation to be comparable or better than standard navigation [6, 12]. Our findings further support two key findings of a study conducted by Brendle et al. [1] comparing a hand-held navigation device with an external screen. First, we also found abstract visualizations to result in significantly lower cognitive load and higher usability ratings compared to standard navigation. Second, when comparing the same visualization either displayed in close proximity (sectional views) or on an external screen (standard navigation), no significant differences were found in cognitive load and ease of use. We therefore conclude that cognitive load and ease of use are mostly affected by the design of a visualization and not its distance.

The trajectory deviations, however, were shown to be affected by the distance of the visualization. While navigating the tool, the eyes must provide the necessary information from the visualization to steer the hand movement. At the same time, the approximately 20% dwell time on the entry point area for visualizations displayed with offset indicate that it is not sufficient to only examine the visualization. Instead, participants also examine the entry point area to coordinate the hand movement, which, in the case of the external screen, requires the eye-gaze to travel much longer distances between screen and tool position. The dwell times further suggest that visualizations generally guide visual attention to where they are displayed. For visualizations displayed at a distance, this results in visual attention being pulled away from the patient, whereas with the peripheral rings the visual attention is actively guided to the area of execution.

For future studies, it would be interesting to investigate how a combination of the most promising anatomical and abstract visualizations, i.e., the virtual twin and the peripheral rings, affect the user experience and preferences. This was also suggested by several participants during the interviews. In addition, our study only examined AR visualizations for accurate alignment of the tool with the intended trajectory. Determining the position of the entry point is an important and challenging step that is also likely to benefit from different AR visualizations. The virtual twin could be particularly interesting, as participants already reported it as useful for understanding the anatomy and for locating the entry point.

Our study setup has several limitations. First, our results were generated in a simulator setup, so further studies are needed to verify these findings in real interventions. While the 490 pre-drilled pedicle screw trajectories were sufficient for statistical analysis of trajectory deviations, a higher number of participants would increase statistical power of questionnaire results, especially as questionnaires are a subjective assessment metric. Furthermore, there may have been a learning curve in working with the physical setup that negatively affected standard navigation results. Allowing participants to operate two sides for standard navigation was aimed at compensating this effect. Despite the fact that the drill sleeve was jagged at the tip, participants sometimes slipped and had to reposition the tool at the entry point. While we do not expect an effect on the trajectory deviations, roughening the model around the entry point area would improve the tool handling for participants. Providing an additional tool for entry point preparation was expected to be unfeasible when used on 35 entry points, given the already complex study setup.

## Conclusion

Using accurate and easy-to-use visualizations for navigation is important to effectively assist surgeons during a procedure. With our work, we contribute a study that investigates the advantages and disadvantages of different AR visualizations by jointly analyzing task performance, visual attention, and user experience. Taking the example of pedicle drilling, the design of AR visualizations has been shown to have a big impact on trajectory deviations, ease of use, and cognitive load. It is therefore important for future AR systems to consider different designs in the development process. When designing anatomical visualizations, it is not necessary to overlay anatomical information on the real anatomy to get a good spatial understanding during navigation. Our results suggest that it is actually easier to combine haptic and visual feedback when anatomical information is displayed with some offset, as the visibility on tool and entry point area are of high importance. In contrast to anatomical visualizations, the main advantage of abstract visualizations lies in the design freedom, such as the possibility to freely adjust the resolution or to fade in the information in the peripheral area of the tool entry point. The latter is particularly promising because visual attention is guided in such a way that information acquisition and coordination of hand movement can take place simultaneously, allowing surgeons to navigate without the need to take their eyes off the patient.
